# Mice recognize 3D objects from recalled 2D pictures, support for picture-object equivalence

**DOI:** 10.1038/s41598-022-07782-4

**Published:** 2022-03-09

**Authors:** Sarah J. Cohen, David A. Cinalli, Herborg N. Ásgeirsdóttir, Brandon Hindman, Elan Barenholtz, Robert W. Stackman

**Affiliations:** 1 Center for Complex Systems and Brain Sciences, Florida Atlantic University, Boca Raton, FL 33431 USA; 2 Jupiter Life Science Initiative, Florida Atlantic University, John D. MacArthur Campus, Jupiter, FL 33458 USA; 3 Department of Psychology, Charles E. Schmidt College of Science, Florida Atlantic University, Boca Raton, FL 33431 USA; 4 FAU and Max Planck Florida Institute Joint Integrative Biology - Neuroscience Graduate Program, Florida Atlantic University, Jupiter, FL 33458 USA

**Keywords:** Long-term memory, Intelligence, Perception, Neural circuits

## Abstract

Picture-object equivalence or recognizing a three-dimensional (3D) object after viewing a two-dimensional (2D) photograph of that object, is a higher-order form of visual cognition that may be beyond the perceptual ability of rodents. Behavioral and neurobiological mechanisms supporting picture-object equivalence are not well understood. We used a modified visual recognition memory task, reminiscent of those used for primates, to test whether picture-object equivalence extends to mice. Mice explored photographs of an object during a sample session, and 24 h later were presented with the actual 3D object from the photograph and a novel 3D object, or the stimuli were once again presented in 2D form. Mice preferentially explored the novel stimulus, indicating recognition of the “familiar” stimulus, regardless of whether the sample photographs depicted radially symmetric or asymmetric, similar, rotated, or abstract objects. Discrimination did not appear to be guided by individual object features or low-level visual stimuli. Inhibition of CA1 neuronal activity in dorsal hippocampus impaired discrimination, reflecting impaired memory of the 2D sample object. Collectively, results from a series of experiments provide strong evidence that picture-object equivalence extends to mice and is hippocampus-dependent, offering important support for the appropriateness of mice for investigating mechanisms of human cognition.

## Introduction

It is often said that a picture is worth a thousand words. Viewing a vacation photo, for example, can elicit full recollection of the when, where, why, and what of the event that occurred at the instant the photo was taken. The picture functions as a representation of that episodic or event memory and can serve to trigger memories of, or related to, the pictured object. In humans, general knowledge about real-world items is often *acquired* in the first place through visual stimuli in 2D form, such as in print media, television, and the Internet. In such formats, the 2D stimuli act as symbols of the actual physical item. For example, we can learn about a monument or landmark, such as the Eiffel Tower, by repeatedly experiencing symbolic representations of it in the media; in doing so, we can deduce its actual structure. Upon our first visit to Paris, the tower is recognized from our memory of the previously viewed images in the media; clearly, pictures are worth a thousand words.

Photographs or pictures representing real-world physical items have traditionally been used to study visual recognition memory in primates, birds, and rodents because such images provide a consistent stimulus presentation regardless of viewing angle or orientation of the subject. However, it is unclear, in all species studied, whether these testing procedures elicit the perceptual inference required to fully relate a pictured object to its 3-dimensional (3D) physical form—a process known as picture-object equivalence^1^. Perceptually, infants can appropriately differentiate a 3D object from that of its 2-dimensional (2D) pictured representation^2,3^, yet the ability to form relationships between symbolic representations and their real-world references follows a developmental arc, which may preclude younger infants from exhibiting representational inference^4,5^. Numerous non-human species can adequately perform 2D picture recognition^6–8^ and true picture-object equivalence has been demonstrated in some nonhuman primates as well as in pigeons^9–11^. However, whether these abilities extend to other species is not well established. Further, the neural circuits and brain mechanisms that support picture-object equivalence have not been determined.

Recent reports demonstrate that rats can perform 2D picture recognition and discrimination^7,12,13^, (for a review see^14^) and that damage to the hippocampus following learning leads to impaired discrimination^15^. Interestingly, both rats and mice demonstrate view-invariant object recognition capabilities, reminiscent of declarative memory processes in human^16^. Results from several studies demonstrate a significant role of the mouse hippocampus in nonspatial *object* recognition memory^17–23^. Specifically, results reveal that mice and rats permitted to explore a novel 3D object for a designated amount of time can accurately recognize the object as being familiar when it is encountered up to 24 h later. The encoding, consolidation and retrieval of the long-term memory for the event of exploring the object is dependent upon hippocampal neuronal activity (for a review, see^24^). However, relating a 2D image from memory to an actual 3D object in view represents a higher-order, flexible use of memory akin to the transitive inference demonstrated in rats^25–28^, in which rats infer the relationship between items that are not presented together, but are directly related.

Based on an extensive review of the literature, it has been suggested that the “representational insight”^29^ required for perceiving that a 2D picture of an object corresponds to its 3D physical form—that is, picture-object equivalence, may be beyond the ability of rodents^1^, or poses substantial problems in experimental design^14^. Therefore, studies of picture-object equivalence have largely overlooked rodents as experimental subjects, in favor of human and nonhuman primates^2,3,9,11^. The preference for higher-order subjects in studies of advanced visual processing of objects may be driven by the traditional notion that rats have low visual acuity, and that rat visual cortex lacks a functional columnar organization typical of cats, tree shrews and primates^30^. However, recent studies suggest that rodents have the capacity for advanced processing of shape and object information^31,32^; for a review, see^33^. Nonetheless, the question of whether rodents are capable of picture-object equivalence remains unanswered.

Here, we tested whether mice could perform tasks with visual perceptual demands like those typically presented to primates and birds in tests of picture-object equivalence. Using several variations on a traditional object recognition task, we tested whether mice could discriminate novel 2D or 3D stimuli based upon prior exposure to their 2D referents, even when presented in different physical forms (e.g., in 2D or 3D form). Overall, we found that if mice spend sufficient time (e.g., 30 s) viewing pictures of an object, then they can subsequently discriminate between a “familiar” 3D physical object and a novel 3D physical object, even when low-level visual strategies are controlled for. Importantly, these findings suggest that after encoding the 2D visual stimuli, mice employ higher-order cognitive processes to associate the 3D item with the recalled memory of the 2D referent. These results provide compelling evidence that mice can spontaneously generalize behavioral responses for viewed pictures of objects to their actual 3D forms, i.e., the behavior of the mice is consistent with picture-object equivalence. In addition, we found that temporary inhibition of neuronal activity in the CA1 region of mouse hippocampus during object memory consolidation eliminated this cognitive ability, indicating that picture-object equivalence in mice requires hippocampal-dependent memory. These results provide support for the view that mice are capable of advanced hippocampal-dependent perceptual capabilities and indicate the appropriateness of mice as models for mechanistic studies of object recognition.

## Results

### Recognition of a 3D object from a 2D picture is hippocampal dependent, regardless of object symmetry

**Figure 1:**
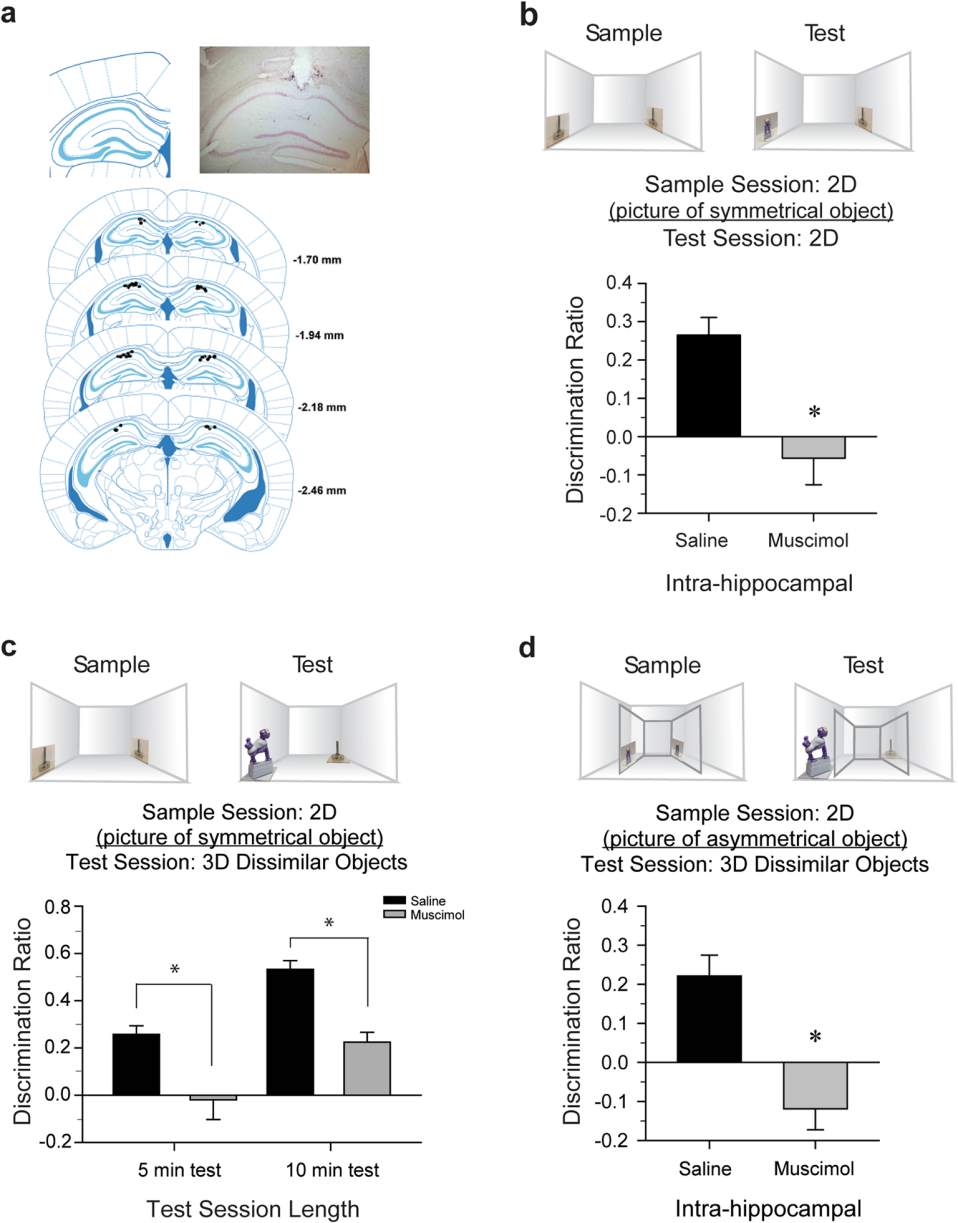
Recognition of a 3D object from a 2D picture is hippocampal dependent regardless of symmetry. (**a**) Representative infusion sites within the CA1 region of dorsal hippocampus and representative photomicrograph of cannula placement (*inset*). (**b**) Each mouse explored two identical novel pictures of a radially symmetric metal leveling foot during sample session (top left). Upon acquiring sample session picture exploration criterion, the mouse was removed and received bilateral intrahippocampal saline or muscimol. During the test session 24 h later (top right), a novel picture replaced one of the familiar pictures. Saline-treated mice explored the novel picture significantly more than the familiar picture during the test session, behavior consistent with visual recognition memory. However, post-sample muscimol-treated mice explored both pictures equivalently, indicating a failure of recognition memory. Discrimination ratio scores were significantly different between the post-sample treatment groups; this difference between the groups was not due to a difference in overall object exploration during the test session. *P < 0.01 versus the respective saline condition. (**c**) The sample session was conducted as in Fig. 1b. During the test session 24 h later (top right), the 2D familiar pictures were replaced with a 3D “familiar” object (i.e., viewed previously in picture form) and a 3D novel object. Saline-treated mice explored the novel object significantly more than the "familiar" during the 5-min test session; behavior consistent with picture-object correspondence. Muscimol-treated mice explored both objects equivalently, implying that hippocampal inactivation impaired memory for the pictured object, and consequently these mice failed to exhibit test session behavior consistent with picture-object correspondence. A second cohort of saline- and muscimol-treated mice explored the novel object more than the familiar when given a 10-min test session. *P < 0.05 versus respective saline condition. (**d**) From within a Plexiglas insert, the sample session was conducted as in Fig. 1b&c; however, the stimuli were pictures of a radially asymmetric monkey. The 5-min test session was conducted as in Fig. 1c. Saline-treated mice explored the novel object significantly more than the "familiar" during the test session; behavior consistent with picture-object correspondence. Muscimol-treated mice explored both objects equivalently, implying that hippocampal inactivation impaired memory for the pictured object, and consequently these mice failed to exhibit test session behavior consistent with picture-object correspondence.

For the inactivation of CA1 neuronal activity experiments, cannulae placements were histologically verified, and analyses included only data from mice with correct placement (Fig. 1a). First, we confirmed that under our conditions, naïve mice could successfully perform a 2D picture recognition memory task and then demonstrated that consolidation of such picture memory was dependent upon neuronal activity from the CA1 region of dorsal hippocampus (Fig. 1b), as previously reported^7,12^. Each mouse explored two identical novel pictures of a radially symmetric cylindrical metal leveling foot during the sample session (Fig. 1b, top left). Upon acquiring the criterion amount of picture exploration during the sample session, the mouse was removed from the test arena and received bilateral intra-CA1 infusions of saline or muscimol. During the test session 24 h later (Fig. 1b, top right), a novel picture replaced one of the familiar pictures. The post-sample saline-treated mice (n = 9) explored the novel picture significantly more than they did the familiar picture during the test session [*t*(8) = − 4.97, *P* < 0.01, *d* = 2.02]; behavior consistent with visual recognition memory. However, post-sample muscimol-treated mice (n = 9) explored both pictures equivalently [*t*(8) = 0.89, *n.s.*], indicating a failure of recognition memory. Discrimination ratio scores were significantly different between the post-sample treatment groups [*t*(16) = 3.85, *P* < 0.01, *d* = 1.81, Fig. 1b]. This difference between the groups was not due to a difference in overall object exploration during the test session [saline 16 s, muscimol 21 s; *t*(16) = − 1.92, *n.s.*].

Secondly, we tested whether mice could spontaneously generalize recognition of actual 3D objects based on their memory of previously viewing a 2D picture of the object. In addition, we tested whether that capacity required hippocampal-dependent memory (Fig. 1c). During the sample session, mice explored 2D pictures of the radially symmetric object (cylindrical leveling foot). During the test session, mice were presented with two 3D objects: the “familiar”, actual physical object that had been presented in picture form during the sample session (foot), and another object that was novel (monkey). Supplementary Fig. S1 depicts a close-up image of the 3D objects used in this and in the following experiments. Mice that received bilateral intra-CA1 infusions of saline immediately after the sample session (n = 9), preferentially explored the novel monkey over the “familiar” foot during the test session [*t*(8) = − 5.63, *P* < 0.01, *d* = 2.28], while those that received intra-CA1 muscimol (n = 11) explored both objects equivalently [*t*(10) = − 0.28, *n.s.*]. Thus, discrimination ratios were found to be significantly different between treatment groups [*t*(18) = 2.82, *P* = 0.01, *d* = 1.32, Fig. 1c], although total object exploration times were equivalent [saline 62 s, muscimol 56 s; *t*(18) = 1.15, *n.s.*]. These results suggest that post-sample inhibition of CA1 neuronal activity impaired memory for the pictured object, and thereby compromised the ability to internally compare the 3D test objects to the sample session pictures. The exploration preference of saline-treated mice for the novel object over the “familiar” one is consistent with picture-object correspondence. When compared with previously published data of saline-treated mice tested in a standard object recognition task (3D objects presented in both the sample and test sessions, or ‘3D-3D’ mice), for data see^17^, 2D-3D and 3D-3D mice exhibited equivalent preference for the novel object [*t*(15) = − 1.04, *n.s.*]. However, time spent exploring the “familiar” object was greater for the 2D-3D mice than that of the 3D-3D mice [*t*(15) = 2.82, *P* = 0.01, *d* = 1.35]. This additional object exploration time may represent the time required to adequately associate the “familiar” 3D object with the recalled memory of the 2D picture. A follow-up 2D-3D experiment was conducted with naïve mice in which the test session was extended to 10 min. Although the duration of the test was extended, the saline-treated (n = 11) and muscimol-treated (n = 9) mice again explored the novel object more than the familiar [*t*(10)  = − 9.91, *P* < 0.01, *d* = 3.93 and *t*(8) = − 5.75, *P* < 0.01, *d* = 1.71, respectively]. Although both groups performed above chance, discrimination ratio scores were significantly lower for the muscimol mice as compared to the saline mice [*t*(18) = 5.56, *P* < 0.01, *d* = 2.50, Fig. 1c]. There was little difference in overall object exploration during the test session [saline 118 s, muscimol 93 s; *t*(18) = 2.43, *P* = 0.03, *d* = 1.10]. Further, an analysis of discrimination ratios, with total exploration as a covariate, preserved the significant treatment effect stated above [ANCOVA: *F*(1,17) = 54.48, *P* < 0.01], *P* < 0.05 versus respective saline condition. Similarly, for 3D-3D mice for 5 min data see^17^, object discrimination improved when the test session duration was extended to 10 min [*t*(19) = − 2.81, *P* = 0.01, *d* = 1.24, Supplementary Fig. S2]. These findings indicate that, unlike other animals^34^, novelty-induced exploration does not diminish over a short period in mice, regardless of the form in which sample stimuli were presented.

The observed successful picture-object recognition by saline control mice could have been a consequence of our pictured sample object having radial symmetry, making it invariant to viewing angle and easier to generalize to the actual object. To test this, we permitted naïve mice to view two identical pictures of a radially asymmetric contoured object from within a clear Plexiglas insert (monkey; Fig. 1d). Since these pictures provide a limited view to the mice of significant shape details of the anterior portion of the asymmetric object, we found in a preliminary study that the physical access to the object’s posterior during the test session, led to greater exploration of this “familiar” object—superseding novelty (see Supplementary Fig. S3a). Eliminating tactile exploration of the objects, by using the clear Plexiglas insert, removed this confound for appropriately assessing picture-object correspondence (for pilot data see Supplementary Fig. S3b). Here, saline-treated mice (n = 9) preferentially explored the novel over the “familiar” object [*t*(8) = − 3.69, *P* < 0.01, *d* = 1.48], while muscimol-treated mice (n = 9) exhibited equal preference [*t*(8) = 1.83, *n.s.*]. Therefore, discrimination ratio scores differed significantly by treatment group [*t*(16) = 4.49, *P* < 0.01, *d* = 2.11, Fig. 1d], yet total object exploration times did not differ [saline 51 s, muscimol 57 s; *t*(16) = − 1.25, *n.s.*]. It is important to also note that the experiments of Fig. 1c, d were essentially a counterbalance of the objects presented during the sample session, yet silencing CA1 neuronal activity impaired consolidation of memory for the explored 2D picture whether it depicted a symmetrical or asymmetrical object.

### Discrimination of an individual object presented in both 2D and 3D forms

**Figure 2:**
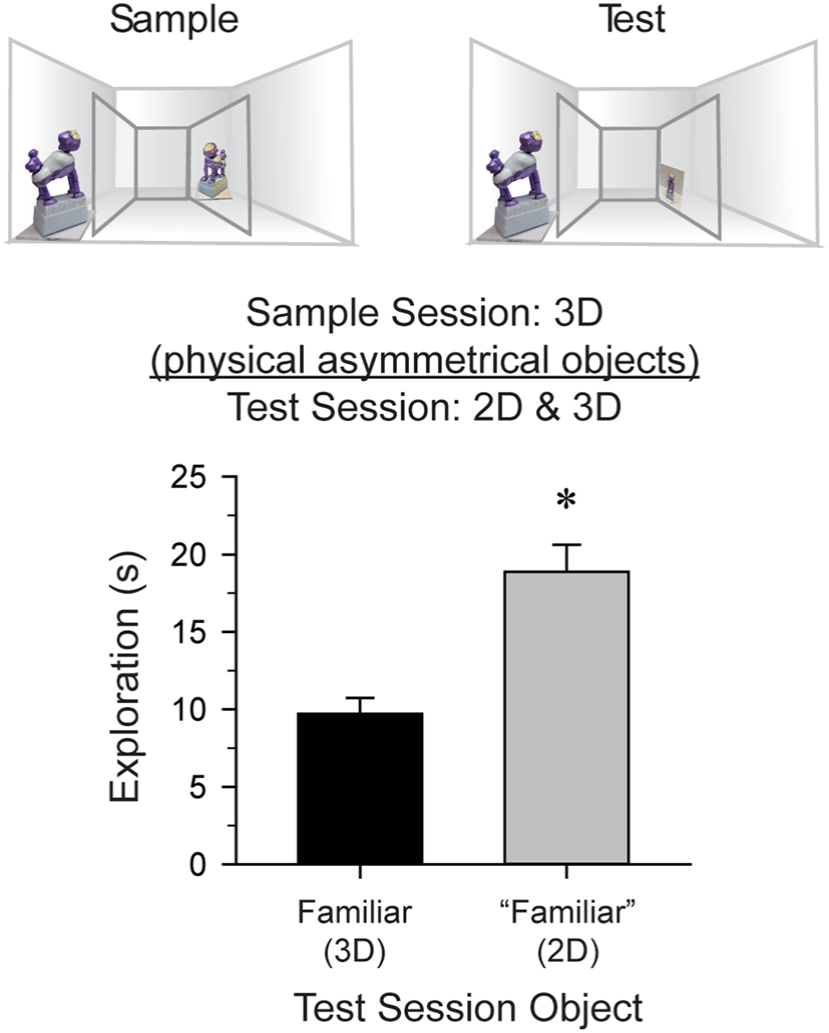
Discrimination of an individual object presented in both 2D and 3D forms. It is possible that limitations of the mouse visual system may preclude mice from truly perceiving the difference between an actual 3D object and a 2D picture of that object. This experiment confirmed that the mice identify the 2D pictures and 3D objects as separate entities, as opposed to perceiving them as the same stimulus. During the sample session, naïve mice (*n* = 9) were placed within the Plexiglas arena insert for a maximum of 10 min where they visually explored two identical 3D objects. During the test session 24 h later, the mice were returned to the insert and allowed 5 min to visually explore the familiar 3D monkey and a 2D picture of the “familiar” monkey. The mice preferentially explored the “familiar” 2D picture over the familiar 3D object [*t*(8) = − 6.79, *P* < 0.01,* d* = 2.13]. This result indicates that rodents identify the 2D “familiar” picture as a stimulus visually distinct from that of the familiar 3D object. *P < 0.01 versus the familiar 3D object.

The interpretation of the above findings is contingent on a mouse’s ability to perceive the difference between an actual object and a picture of that same object, as opposed to picture processing in a *confusion* mode (in which 2D and 3D stimuli are viewed as the same entity^35^). To test this, mice (n = 9) visually explored the two identical 3D monkey objects, and then one monkey object was replaced with a picture of that “familiar” monkey for the test session. Object discrimination [*t*(8) = 7.80, *P* < 0.01, *d* = 2.60] with preferential exploration of the “familiar” picture [*t*(8) = − 6.79, *P* < 0.01, *d* = 2.13, Fig. 2] indicated that mice perceive a physical object and a picture of the identical object as inherently different stimuli.

### Recognition of a 3D object from a 2D picture is not affected by low-level visual properties or viewing angle

**Figure 3:**
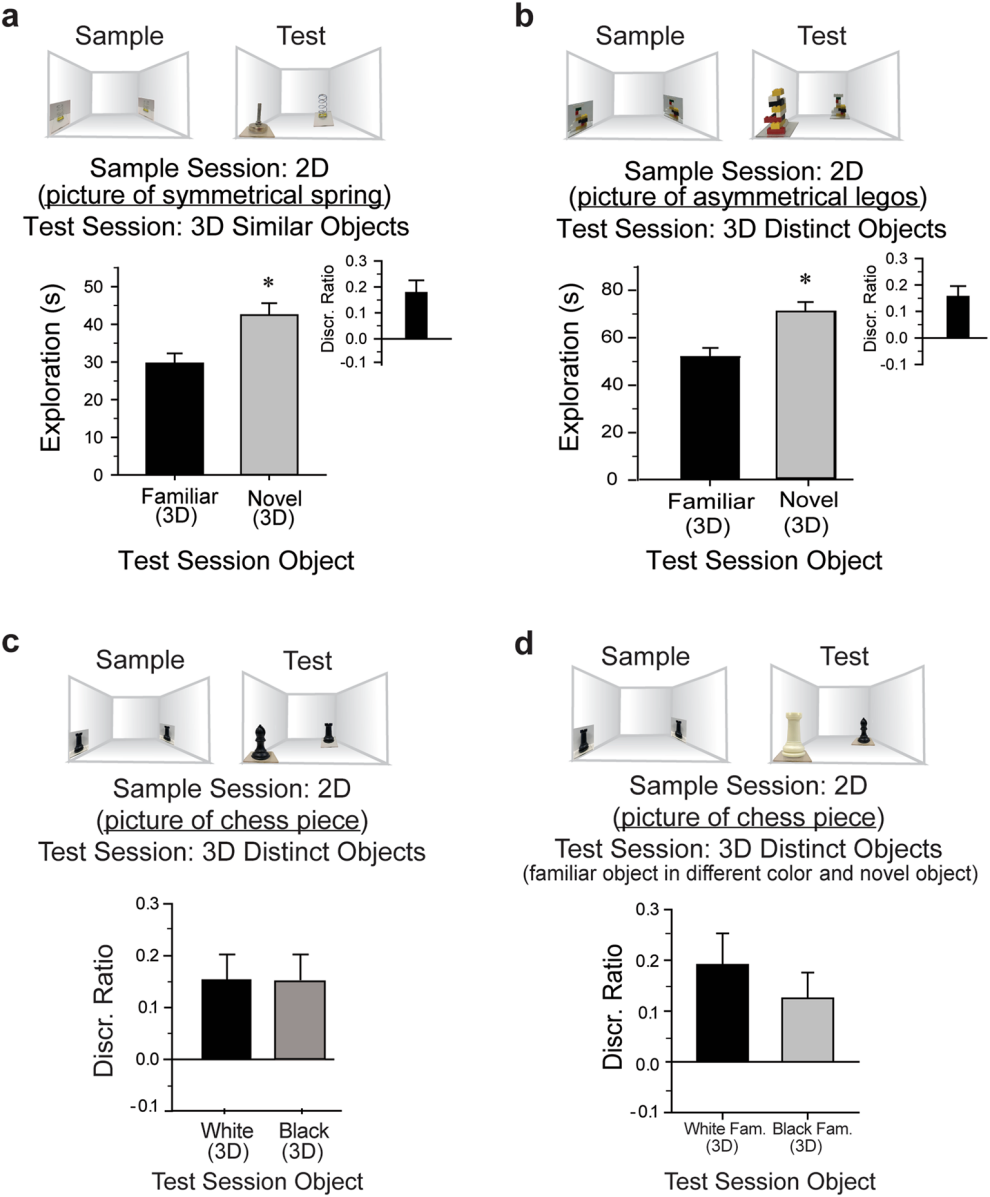
Recognition of a 3D object from a 2D picture is not affected by low-level visual properties. (**a**) During the sample session, mice viewed 2D pictures of a stimulus visually similar to the test session novel object. During the test session 24 h later, the mice explored the “familiar” object significantly less than the novel object. Inset, mice exhibited a significant discrimination of the novel object over the “familiar” object. (**b**) During the sample session, mice viewed 2D pictures of a stimulus visually dissimilar to the test session novel object in configuration only. During the test session 24 h later, the mice explored the “familiar” object significantly less than the novel object. Inset, mice exhibited a significant discrimination of the novel object over the “familiar” object. (**c**) To account for low-level luminance differences that may be guiding novel object preference, mice viewed 2D pictures of a chess piece (rook or bishop that were either black or white in color) during the sample session. During the test session 24 h later, mice exhibited a significant discrimination of the novel object over the “familiar” object regardless of the color in which the objects were presented. (**d**) During the sample session, mice viewed 2D pictures of a chess piece (rook or bishop that were either black or white). During the test session 24 h later, mice entered the familiar arena with the ‘familiar’ object in a novel color (i.e., if the mice received a picture of the white rook during sample, then during test, the rook was black) and the novel object in the same color as the sample 2D pictures. Regardless of the color in which the object was represented in the sample pictures, the mice preferentially explored the novel object and there was no difference in discrimination performance as a result of the color in which the stimuli were presented.

A subsequent picture-guided object discrimination experiment revealed that even under conditions in which the objects only differed in terms of color and configuration, mice (n = 10) exhibited a preference for the test-session novel object (foot and spring; Fig. 3a; *t*(9)  = − 3.85, *P* < 0.01,* d* = 2.57). Evidently, mice are able to discriminate between test session “familiar” and novel objects regardless of visual similarities [*t*(9) = 3.71, *P* < 0.01, *d* = 1.17, Fig. 3a]. Further, to account for differences in salient low-level features, mice (n = 9) were tested using asymmetric objects that were of similar size, color, and luminance, but differed in configuration (toy LEGO constructions, see Fig. 3b top). However, even under these conditions, mice continued to maintain preference for the novel test object (*t*(8) = − 4.38, *P* < 0.01, *d* = 2.06; Fig. 3b), and were clearly able to discriminate between test session stimuli that were indistinguishable aside from shape [*t*(8) = 4.68, *P* < 0.01, *d* = 1.56, Fig. 3b]. To further test whether low-level features such as color and luminance of the 2D stimuli guide novel 3D object preference, naïve mice (n = 16) were tested using similar sized plastic chess pieces (rook and bishop) presented in either white or black. Regardless of color, mice continued to prefer the novel object during the test session (white pieces: *t*(7) = 3.670, *P* < 0.01, *d* = 1.23; black pieces: *t*(7) = 2.468, *P* < 0.04, *d* = 1.03; Fig. 3c). Importantly, there was no significant test session discrimination difference between the mice that received white versus black chess pieces as their sample and test stimuli [*t*(14) = 0.136, *n.s.*, Fig. 3c]. A variation of this experiment was also conducted in which the test session ‘familiar’ object was presented in the opposing color to its presentation during the sample session and the novel object was presented in the same color as the sample stimuli. In this case, regardless of the color in which the object was represented in the sample pictures, the mice preferentially explored the novel object [white objects for sample and novel object: *t*(7) = 3.068, *P* = 0.01, *d* = 2.56; black objects for sample and novel object: *t*(7) = 2.475, *P* = 0.04, *d* = 1.82; Fig. 3d]. Importantly, there was no difference in discrimination performance as a result of the color in which the stimuli were presented [*t*(14) = 0.82, *n.s.*, Fig. 3d]. This result suggests that when controlling for differences in color, contrast, or luminance, mice are still able to correctly associate the recalled memory of the 2D picture to the actual 3D object, enabling it to be identified as “familiar”, which then resulted in preferential exploration of the novel object. Although beyond the scope of the present report, further studies will be needed to determine the degree to which neuronal activity in lower-level visual cortical areas are sufficient to support visual object recognition based on luminance, as has been shown in rats^36^.

**Figure 4:**
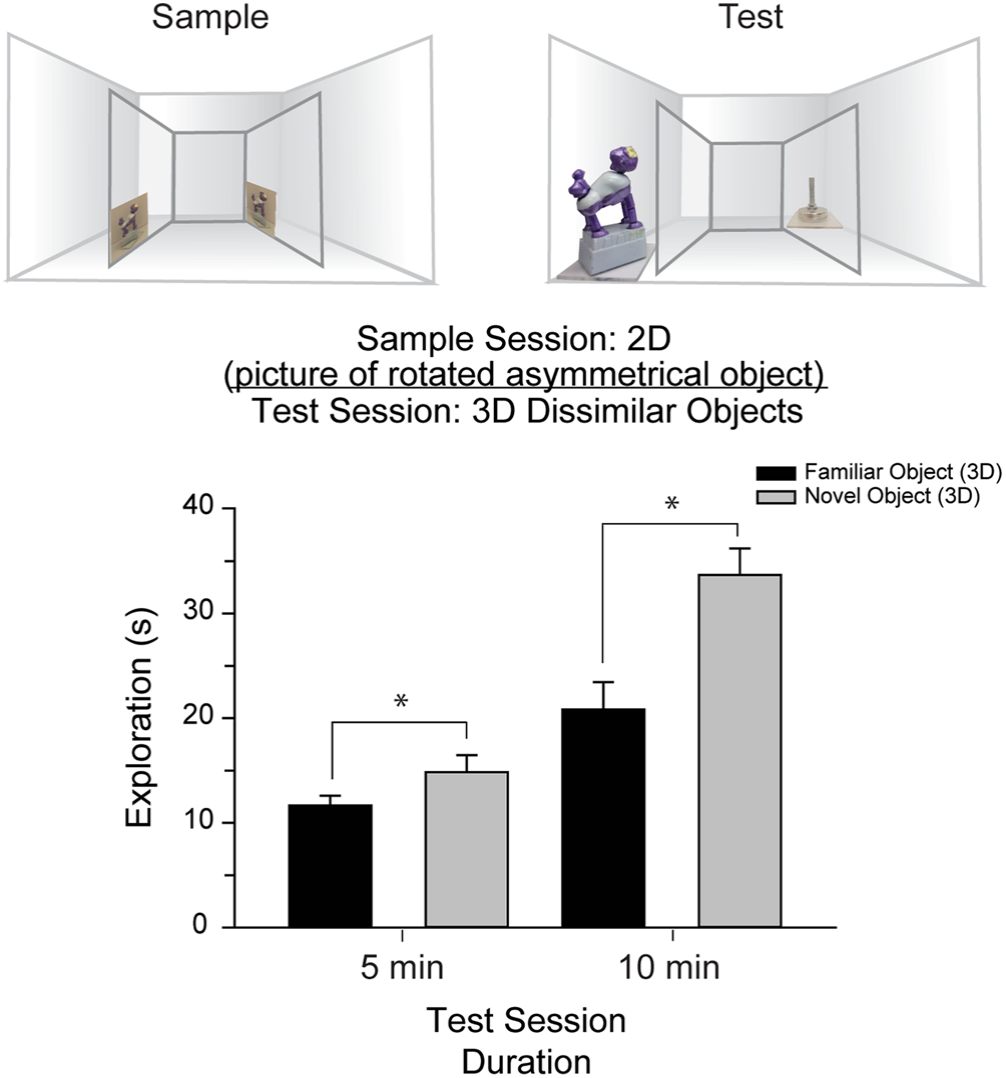
Recognition of a 3D object from a 2D picture is not affected by viewing angle. During the sample session, mice viewed pictures depicting the monkey in a rotated or profile view from within the Plexiglas insert. During a 5-min test session 24 h later, mice explored the novel object significantly more than the (non-rotated) “familiar” object. Next, we tested whether extending the duration of the test session would affect the expression of picture-object equivalence in mice. Naïve mice (n = 10) explored pictures of the monkey in a rotated or profile view during the sample session. During a 10-min test session 24 h later, the mice exhibited a significant preference for the novel 3D object over the “familiar” monkey [*t*(9) = − 5.12, *P* < 0.01, *d* = 1.60]. Inset, mice exhibited a significant discrimination of the novel object over the “familiar” object [*t*(9) = 4.86, *P* < 0.01, *d* = 1.54]. Compared to the object discrimination elicited with a 5-min test session, extending the duration of the test session increased object discrimination [*t*(18) = − 2.54, *P* = 0.02, *d* = 1.14]. Thus, this experiment also indicates that mice do not lose their proclivity to explore novel items after short intervals of time. *P < 0.05 versus 5-min test.

**Figure 5:**
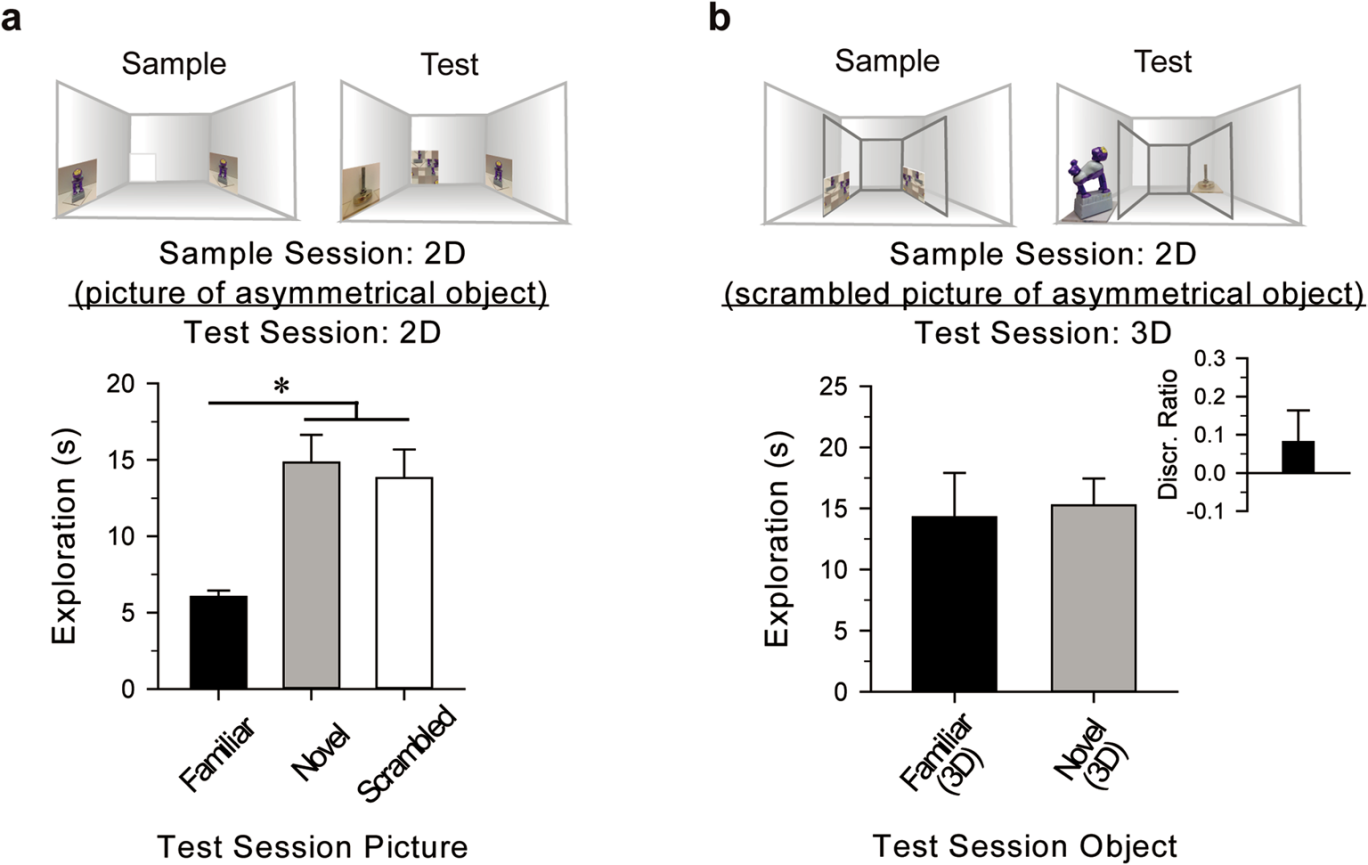
Mice rely on composite images for subsequent object recognition. (**a**) Mice are incapable of matching scrambled images of an object to its actual 3D form or its holistic image. During a sample session, mice explored pictures of an asymmetric object, and a blank picture. During a test session 24 h later, the mice explored both the novel picture and scrambled picture of the "familiar" object significantly more than the familiar picture. This result suggests that mice likely recall the sample session picture as a composite image. (**b**) Mice explored pictures of a scrambled object from within the Plexiglas insert, then 24 h later, visually explored the “familiar” and the novel objects equivalently. Inset, mice did not discriminate between test objects, indicating that accurate identification of the “familiar” object is only possible when the picture representation is sufficiently similar to the actual object. *P < 0.05 versus familiar picture.

Further, in another version of the task, rather than presenting pictures of the asymmetrical monkey, mice (n = 10) were presented pictures of the monkey in a rotated or side profile view during the sample session (Fig. 5). During the test session, the mice discriminated between the “familiar” monkey, now presented from a different view than that of the sample picture, and the novel foot [*t*(9) = 4.07, *P* < 0.01, *d* = 1.29], with preference for the foot [*t*(9) = − 3.51, *P* < 0.01, *d* = 0.77, Fig. 5]. Doubling the duration of the test session increased object discrimination [*t*(9) = 4.86, *P* < 0.01, *d* = 1.54], and novel object preference [*t*(9) = − 5.12, *P* < 0.01, *d* = 1.60, Fig. 5]. Compared to the 5-min test, mice in the extended test session (n = 10) demonstrated greater object discrimination [*t*(18) = − 2.54, *P* = 0.02, *d* = 1.14]. These results suggest that although some visual information about the monkey was absent from the sample picture, mice recall the remembered image and then associate it with the 3D object during the test session. However, it is possible that the mice use low-level visual features, such as luminance or contrast, to correctly associate the 2D picture with the ‘familiar’ 3D object. Although it is difficult to completely rule out recognition based on low-level features using solid objects^14^, the results summarized here are consistent with the mice recognizing a 3D object based on a recalled memory of a previously explored 2D picture.

### Picture/object recognition cannot be supported by only low-level visual features

**Figure 6:**
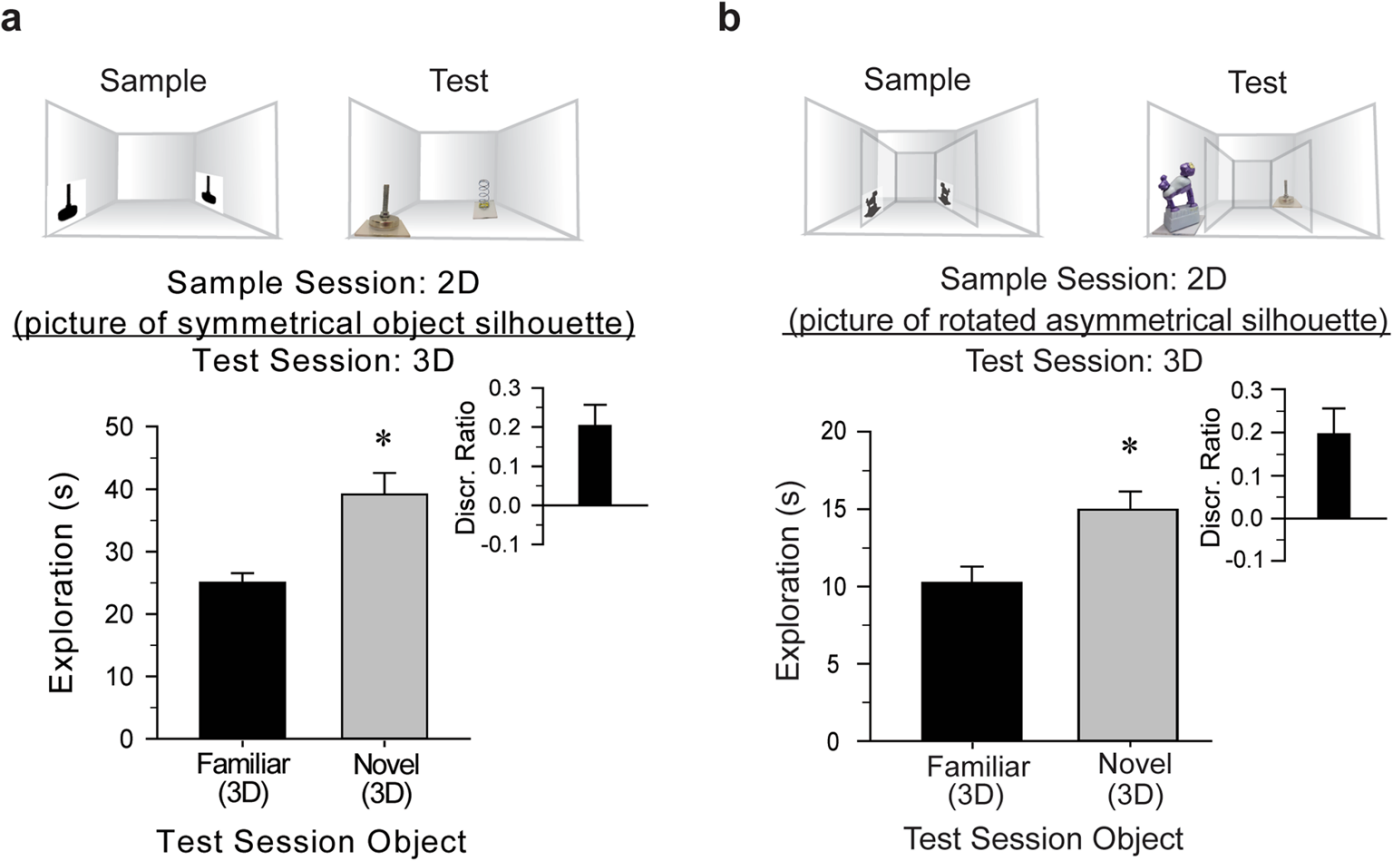
Evidence of picture-object equivalence in mice. (**a**) During a sample session, mice explored abstract silhouette pictures of a symmetrical object, and 24 h later, explored the novel object significantly more than the “familiar” object. Inset, mice demonstrate object discrimination, indicating that the retrieved memory of the viewed silhouette conveyed enough information to permit recognition of the “familiar” object; furthering support for picture-object equivalence in mice. (**b**) Mice explored abstract silhouette pictures of a rotated asymmetrical object, and 24 h later, explored the novel object significantly more than the “familiar” object. Inset, mice demonstrate object discrimination, indicating that the retrieved memory of the viewed silhouette conveyed enough information to permit recognition of the “familiar” object^16^; furthering support for picture-object equivalence in mice. *P < 0.05 versus “familiar” object.

To determine if the recognition reported above reflected the memory of low-level visual features of the sample pictured object (i.e., its colors, contrast or contours), or the memory of the composite and realistic sample image, mice explored pictures of the monkey (familiar) and a blank white picture during a sample session. Twenty-four h later, mice (n = 10) were presented the familiar picture, a scrambled version of the familiar picture, and a novel picture during a test session. The novel and scrambled pictures were preferentially explored over the familiar [*F*(2, 27) = 10.03, *P* < 0.01, η^2^ = 0.43 Fig. 6a], with preferential exploration of the novel over the familiar (*P* < 0.01), the scrambled over the familiar (*P* = 0.01), and equivalent exploration of the novel and scrambled*.* These results are consistent with the notion that preferential exploration of novel test stimuli is guided by memory of the realistic image, rather than its scrambled individual features.

Complementary to the previous experiment, mice (n = 8) that explored pictures of the scrambled monkey, failed to preferentially explore either the “familiar” monkey object or the novel foot object during the test session [*t*(7) = − 0.35, *n.s.*, Fig. 6b], indicating non-discrimination [*t*(7) = 0.98, *n.s.*, Fig. 6b]. These results further suggest that mice cannot recognize objects that were previously viewed in picture form if the sample stimuli are not presented as realistic representations of the test object. Consistent with the findings using the toy LEGOs and chess pieces, these results suggest that discrimination of 3D objects based on learned information from 2D pictures is not simply dependent on low-level visual feature similarity or luminance. However, rats have been shown to use low-level visual features, such as luminance, to discriminate between 2D shapes^37,38^.

### Evidence of picture-object equivalence: item recognition from a remembered silhouette or outline

Matching a depicted abstract object to the actual object is a strong demonstration of cognition and equivalence. Similar to procedures used in previous studies of higher-order species^39^, we tested item recognition when the pictures represented more realistic images of the “familiar” object, yet were lacking low-level features (e.g., a silhouette). Mice (n = 10) that explored silhouette images of the foot during a sample session, accurately discriminated between the “familiar” foot and the novel spring during the test session [*t*(9) = 3.74, *P* < 0.01, *d* = 1.18, Fig. 6a *inset*], preferentially exploring the spring [*t*(9) = − 4.17, *P* < 0.01, *d* = 1.67, Fig. 6a]. Further, mice that explored silhouettes of the asymmetric monkey during a sample session, accurately discriminated between the test session “familiar” monkey and novel foot [*t*(9) = 3.25, *P* = 0.01, *d* = 1.03, Fig. 6b *inset*], demonstrating a preference for the foot [*t*(9) = 19.79, *P* < 0.01, *d* = 6.26, Fig. 6b]. Similarly, mice that were presented with 2D pictures of an outlined object during the sample session and received the ‘familiar’ 3D object and novel 3D object during the test session, preferentially explored the novel object, indicated recognition of the ‘familiar’ object (see Supplementary Fig. S4). These results are consistent with evidence that rats can use shape information to support 2D object recognition^31,37,38,40^, and suggest that learned information about the 2D sample items can support subsequent recognition of the object in its 3D form. By employing a modified version of a primate task, our results suggest that mice establish perceptual correspondence between pictorial representations of objects and their physical forms, behavior consistent with picture-object equivalence.

## Discussion

We sought to determine whether rodents, which serve as an important animal model of cognition, are capable of picture-object equivalence. Specifically, we tested whether mice could make inferential judgments between an object and a recalled memory of a picture depicting that same object. Our findings that mice preferentially explore a novel over a “familiar” object (previously experienced in picture form) imply that mice exhibit picture-object equivalence. Further, results from control studies demonstrate evidence for this picture-object equivalence regardless of object symmetry, likeness, viewing angle, composition, and image realism. Even when low-level visual features, such as color and luminance are controlled for (e.g., our chess piece experiments), mice are still able to generalize from 2D picture to 3D object. Importantly, we confirmed that mice perceive the inherent difference between a picture of an object and the actual 3D object itself. Further, we found that picture memory, required for such higher-order inference, depends upon neuronal activity in the CA1 region of dorsal hippocampus. Recognition memory is well established in mice, yet our results extend that literature to indicate that rodents are capable of advanced visual recognition and learn indirectly about actual objects by viewing images^16^. Additionally, consistent with studies in non-human primates^39^, results of the silhouette and outline experiments confirm that mice recognize a 3D object from a retrieved memory of it presented in abstract picture form, providing a compelling demonstration of picture-object equivalence in mice. Of course, the extent to which mice, and other animals, are able to fully conceptualize that the image is a pictorial representation of a 3D object remains unclear^8^. Yet, we provide compelling evidence that regardless of the strategy mice employ to encode visual information about the 2D and 3D stimuli, it is only through a higher-order perceptual process that mice can generalize the learned information about a 2D stimulus to its 3D object form. Furthermore, additional investigation will be required to determine the neural mechanisms that underlie symbolic representations in the mouse brain.

There are two primary ways to conduct these picture-object equivalence studies. In one scenario, the sample session stimuli could have consisted of 3D objects, and the test session of 2D pictures of objects. However, we chose the alternate scenario in which the sample session consisted of 2D pictures, and the test session consisted of 3D objects. We contend that restricting the mouse’s view of the object during the sample session to a single angle afforded by the picture, placed a greater demand on the mice to subsequently relate that recalled picture information to the actual object viewable from multiple angles during the test session. Additionally, this more complex experimental design is aligned with those used in studies of picture-object equivalence in primates^39,41^. The ability of the mice to successfully achieve even this more difficult level of equivalence provides important support for the utility of rodents as animal models for higher-level cognitive processes in humans.

Prevailing views state that object recognition memory is supported by object *familiarity*, which is dependent upon the perirhinal cortex^42,43^, or by object or object-in-context *recollection*, which is dependent upon the hippocampus^17,24,44,45^. Our results demonstrate that visual recognition and picture-object generalization were both impaired in mice that had received post-sample local infusions of muscimol into the CA1 region of hippocampus. These results are consistent with the notion that the hippocampus contributes to nonspatial aspects of declarative or explicit memory^46–48^. We further speculate that our task design assessed hippocampal-dependent object familiarity, provided we define familiarity as recalling the object presented based on retrieval of a stored representation of that object viewed from a single angle in picture form. Although there is likely a complementary role of several brain regions involved in such a complex cognitive function^22^, our results suggest that familiarity with visual stimuli involves the hippocampus as well as the perirhinal cortex^49^.

Given that exploration in our task was spontaneously elicited by the 2D and 3D stimuli, it is difficult to levy alternative explanations for our findings. The results of the present study support the view that mice can perform a higher-order form of visual perception and cognitive abilities, typically associated with primates. Specifically, the ability of mice to make perceptual and conceptual judgments about presented stimuli is surprising given that picture-object equivalence had been considered a defining capacity of human and non-human primates. In primates, the hippocampus is thought to play an essential role in declarative or explicit memory enabling an individual to replay a “story” of a previously encoded experience. We suggest that that “story” enables one to recognize items learned in picture form when they are subsequently presented in 3D form. The mouse hippocampus likely encodes and consolidates the picture exploration as a “story” of that experience or event, within a specific context as a form of explicit memory. Our findings provide strong support that a functional mouse hippocampus is required for this form of nonspatial visual recognition memory and picture-object equivalence, consistent with our previous work and that of others^7,12,17,22,23,50^. The role of the hippocampus may be to retrieve the memory of the picture explored during the sample session, against which the mouse can appropriately match to one of the items available during the test session. Additionally, we provide the first evidence that mice make perceptual and conceptual judgments about presented task stimuli, which is surprising given that picture-object equivalence has been considered a defining capacity of primates. Taken together, our results provide convincing evidence that the mouse may serve as an effective model organism to investigate higher-order sophisticated aspects of mammalian visual perception and recognition.

## Methods

### Mice and surgery

Male C57BL/6J mice (7–10 weeks old; Jackson Labs) were housed 4 per cage with ad libitum access to food and water. All procedures were conducted, and results were reported, in accordance with NIH and ARRIVE guidelines, and were approved by Florida Atlantic University’s IACUC. For all inactivation experiments, surgical implantation of guide cannulae (n = 60) was completed one week after acclimatization to the vivarium. For all other experiments, mice (n = 140) began testing at 8 weeks old, after one week of vivarium acclimatization. Naïve mice were used for each new experiment to ensure all mice were matched for prior testing experience. A sample size of approximately 8–12 mice per treatment group was determined a priori to give us 80% power to detect a moderately sized effect at a significance level of 0.05.

### Surgical cannulation and microinfusion

For the inactivation experiments (Fig. 1 and Supplementary Fig. S2), mice were implanted with chronic bilateral guide cannulae (Plastics One, Inc., Roanoke, VA) above the CA1 region of dorsal hippocampus (A/P—2.0 mm, M/L ± 1.5 mm, D/V—1.1 mm from bregma; corresponding to intermediate CA1), as previously described^17^. Behavioral testing began 7–10 days later to permit postoperative recovery. Each mouse received a “mock infusion” each day for 2 days, immediately after the arena habituation, to acclimate the mice to the microinfusion procedure, as previously described^17^. For the actual microinfusions, mice received bilateral (0.35 µl/side, 0.33 µl/min) intra-hippocampal muscimol (Tocris, 1 µg/µl in 0.9% sterile saline) or 0.9% sterile saline immediately after the sample session. For the actual bilateral microinfusion procedures see^17^. Mice were randomly assigned to treatment groups, regardless of their performance during behavior testing. In addition, the experimenters conducting behavioral testing were blind to treatment groups.

### Object recognition task and protocols

For all experiments, the apparatus consisted of two open-top, high-walled square arenas made of white ABS (each: 37.5 × 37.5 × 50 cm). For the experiments represented in Figs. 1D, 2, 5, 6B, 7B and Supplementary Fig. S3, the mice were restricted to a zone delineated by a clear acrylic square arena insert (each: 25.4 × 25.4 × 50 cm) throughout all stages of testing (including all habituation sessions). The function of the insert was to limit the viewing angle of the stimuli and to remove the ability of the mice to physically interact with the stimuli. During days 1 and 2, each mouse explored one of the arenas during a 10-min empty arena habituation session. On days 3 and 4, each mouse received one sample session and one test session, respectively, in the habituated (i.e., familiar) arena. During the sample session, each mouse was returned to the familiar arena that now contained two identical novel 3D objects (stainless steel cabinet leveling foot, or plastic toy gorilla, or a stainless-steel spring, or toy LEGO constructions, or plastic chess pieces (Chess House, Inc., Lynden, WA), each attached to a Plexiglas base), or two 2D pictures of those same objects (all stimuli were of similar size). Supplementary Fig. S1 provides a close-up view of the 3D objects. The 3D objects were placed on the floor in the NW and SE corners. The two pictures were positioned on the arena walls, 2 cm from the floor (NW and SE). Picture/object exploration was defined as any time the mouse spent with its head oriented toward and within 2–3 cm of the stimuli. Each mouse was removed from the arena upon accumulating a minimum of 30 s exploration of each object/picture or 38 s on either object/picture or 10 min had elapsed; prior experimentation suggested that these exploration times would result in strong object discrimination during the test session 24 h following sample^17,23,51^. Mice were only included in the study if they successfully reached this criterion of exploration during the sample session. This *sample object exploration criterion* was imposed to ensure that all mice were matched for sample session performance and to support a strong and long-lasting memory. Pictures/object stimuli were fully counterbalanced within each experiment, or all picture/object stimuli were previously determined to elicit equal preference by naïve mice. The data from ten mice that failed to reach the sample session exploration criteria within the 10-min session were removed from the analyses. During the test session, presented 24 h later, the familiar arena contained combinations of familiar or novel 2D pictures along with familiar or novel 3D physical representations of 2D stimuli (see “Experiment Outline”below for specific details). The mouse was removed from the arena after either 5 or 10 min (Figs. 1c, 5, and Supplementary Fig. S2). The pictures, objects, and arena (floor, walls and insert) were cleaned with 10% ethanol after each session to remove any olfactory cues. At the completion of each testing day, the pictures, objects, and arena (floor, walls and insert) were cleaned with Vimoba disinfectant (Quip Laboratories Inc., Wilmington, DE). All behavioral testing data was digitally acquired by the EthoVision XT (Noldus Inc., Leesburg, VA) software package. Object exploration was scored off-line from the digital video files by experimenters that were blind to the treatment condition of the mice. Object memory was inferred from test session stimuli exploration differences and the discrimination ratio—calculated for each mouse by subtracting the time spent exploring the familiar picture/object from the time spent exploring the novel picture/object and dividing the result by the total time spent exploring both items (Discrimination Ratio = T_novel_ − T_familiar_/T_novel_ + T_familiar_). Discrimination ratio scores range from − 1 to 1, with positive scores indicating novel stimulus preference, while a ratio = 0 indicating chance performance or a lack of preference for one stimulus over another.

### Experiment outline

The following provides an overview of the different procedures and rationale of each experiment described:

Figure 1b: Confirmation that mice successfully perform picture-picture, or 2D to 2D, stimulus recognition in our testing apparatus and conditions; and to test if 2D to 2D recognition is dependent on hippocampus.

Figure 1c: To test whether mice could spontaneously generalize recognition of 3D *symmetrical* objects based on their memory of previously viewing a 2D picture of the object; and to test whether this function is hippocampal dependent.

Figure 1d: To test whether mice could spontaneously generalize recognition of 3D as*ymmetrical* objects based on their memory of previously viewing a 2D picture of the object; and to test if this function is hippocampal dependent.

Figure 2: To test whether mice can perceive the difference between an actual 3D object and a picture of that same object.

Figure 3a–d: Three experiments testing whether mice can generalize from 2D pictures to 3D objects when low-level visual information is limited by using objects that are different only in color, configuration, or luminance. The key experiments are depicted in Fig. 3c, d in which the only difference between the stimuli is shape (color and luminance are controlled).

Figure 5: To test whether mice can generalize from 2D pictures to 3D objects when viewing angle of the stimuli are different between the sample and test session. Note that although the results suggest mice successfully generalized from the 2D to 3D stimuli, it is possible that the recognition was guided by mere low-level visual features.

Figure 6a, b: Similar test as in Fig. 5, to test whether mice use luminance or other low-level features to discriminate between novel and familiar stimuli.

Figure 6a, b (and Supplementary Fig. 4): Testing 2D picture to 3D object generalization using silhouettes (Fig. 6a, b) and outlines (Supplementary Fig. S4) of the stimuli presented during the respective sample session. The objective in these experiments, was to control low-level visual strategies that mice could employ to aid recognition.

### Data analysis

All data sets were confirmed to be normally distributed, thus permitting parametric statistical analyses. The outcome measures, discrimination ratio and latency (in s) to reach the sample object exploration criterion of saline- and muscimol-treated mice, were compared using two-tailed Student’s *t* tests. Paired *t* tests were used to compare exploration times of each test session stimulus, and one-sample *t *tests were used to compare the discrimination ratios of the respective groups of mice to chance performance. Inter-experimental differences in discrimination ratio were assessed using two-tailed Student’s *t* tests. For Fig. 6a, respective picture exploration times were analyzed by within-subjects ANOVA, followed by post-hoc Bonferroni pairwise comparisons using Holm-Sidak confidence-interval adjustments. For Supplementary Fig. S2, discrimination ratios were analyzed by within-subjects ANCOVA, with total exploration as a covariate. Significant findings were further evaluated using Cohen’s d or η^2^ calculations to determine effect size estimates.

### Histology

Each mouse that had received intra-hippocampal microinfusions was deeply anesthetized with 5% isoflurane at the conclusion of the respective experiment, and brains were dissected and preserved in 4% paraformaldehyde. Cannulae placements were confirmed by examination of cresyl violet-stained 50 µm coronal brain sections under a light microscope (see Fig. 1a). Data for any mice determined to have inappropriately placed cannulae were excluded from the analyses (n = 6).

## Supplementary Information

Supplementary Information 1.

Supplementary Information 2.

Supplementary Information 3.

Supplementary Information 4.

Supplementary Information 5.

## References

[1] Bovet D, Vauclair J (2000). Picture recognition in animals and humans. Behav. Brain Res..

[2] Bower TG (1972). Object perception in infants. Perception.

[3] Hochberg J, Brooks V (1962). Pictorial recognition as an unlearned ability: A study of one child's performance. Am. J. Psychol..

[4] Pierroutsakos SL, Deloache JS, Gound M, Bernard EN (2005). Very young children are insensitive to picture-but not object-orientation. Dev. Sci..

[5] DeLoache JS (2010). Do babies learn from baby media?. Psychol. Sci..

[6] Lubow RE (1974). High-order concept formation in the pigeon. J. Exp. Anal. Behav..

[7] Prusky GT, Douglas RM, Nelson L, Shabanpoor A, Sutherland RJ (2004). Visual memory task for rats reveals an essential role for hippocampus and perirhinal cortex. Proc. Natl. Acad. Sci. U. S. A..

[8] Judge PG, Kurdziel LB, Wright RM, Bohrman JA (2012). Picture recognition of food by macaques (*Macaca silenus*). Anim. Cogn..

[9] Savage-Rumbaugh ES, Rumbaugh DM, Smith ST, Lawson J (1980). Reference: The linguistic essential. Science.

[10] Watanabe S (1993). Object-picture equivalence in the pigeon: An analysis with natural concept and pseudoconcept discriminations. Behav. Proc..

[11] Rosenfeld SA, Van Hoesen GW (1979). Face recognition in the rhesus monkey. Neuropsychologia.

[12] Clark RE, Reinagel P, Broadbent NJ, Flister ED, Squire LR (2011). Intact performance on feature-ambiguous discriminations in rats with lesions of the perirhinal cortex. Neuron.

[13] Forwood SE, Bartko SJ, Saksida LM, Bussey TJ (2007). Rats spontaneously discriminate purely visual, two-dimensional stimuli in tests of recognition memory and perceptual oddity. Behav. Neurosci..

[14] Zoccolan D, Di Filipppo A, Ennaceur A, de Souza Silva MA (2018). Methodological approaches to the behavioural investigation of visual perception in rodents. Handbook of Object Novelty Recognition Handbook of Behavioral Neuroscience.

[15] Epp J (2008). Retrograde amnesia for visual memories after hippocampal damage in rats. Learn. Mem..

[16] Mitchnick KA (2018). Development of novel tasks for studying view-invariant object recognition in rodents: Sensitivity to scopolamine. Behav. Brain Res..

[17] Cohen SJ (2013). The rodent hippocampus is essential for nonspatial object memory. Curr. Biol..

[18] Hammond RS, Tull LE, Stackman RW (2004). On the delay-dependent involvement of the hippocampus in object recognition memory. Neurobiol. Learn. Mem..

[19] Manns JR, Eichenbaum H (2009). A cognitive map for object memory in the hippocampus. Learn. Mem..

[20] Stackman RW, Cohen SJ, Lora JC, Rios LM (2016). Temporary inactivation reveals that the CA1 region of the mouse dorsal hippocampus plays an equivalent role in the retrieval of long-term object memory and spatial memory. Neurobiol. Learn. Mem..

[21] de Lima MN, Luft T, Roesler R, Schroder N (2006). Temporary inactivation reveals an essential role of the dorsal hippocampus in consolidation of object recognition memory. Neurosci. Lett..

[22] Asgeirsdottir HN, Cohen SJ, Stackman RW (2020). Object and place information processing by CA1 hippocampal neurons of C57BL/6J mice. J. Neurophysiol..

[23] Cinalli DA, Cohen SJ, Guthrie K, Stackman RW (2020). Object recognition memory: Distinct yet complementary roles of the mouse CA1 and perirhinal cortex. Front. Mol. Neurosci..

[24] Cohen SJ, Stackman RW (2015). Assessing rodent hippocampal involvement in the novel object recognition task. A review. Behav. Brain Res..

[25] Davis H (1992). Transitive inference in rats (*Rattus norvegicus*). J. Comp. Psychol..

[26] Dusek JA, Eichenbaum H (1997). The hippocampus and memory for orderly stimulus relations. Proc. Natl. Acad. Sci. U. S. A..

[27] Fortin NJ, Agster KL, Eichenbaum HB (2002). Critical role of the hippocampus in memory for sequences of events. Nat. Neurosci..

[28] Van Elzakker M, O'Reilly RC, Rudy JW (2003). Transitivity, flexibility, conjunctive representations, and the hippocampus. I. An empirical analysis. Hippocampus.

[29] Aust U, Huber L (2010). Representational insight in pigeons: Comparing subjects with and without real-life experience. Anim. Cogn..

[30] Hendry SH, Reid RC (2000). The koniocellular pathway in primate vision. Annu. Rev. Neurosci..

[31] Brooks DI (2013). Categorization of photographic images by rats using shape-based image dimensions. J. Exp. Psychol. Anim. Behav. Process.

[32] Djurdjevic V, Ansuini A, Bertolini D, Macke JH, Zoccolan D (2018). Accuracy of rats in discriminating visual objects is explained by the complexity of their perceptual strategy. Curr. Biol..

[33] Zoccolan D (2015). Invariant visual object recognition and shape processing in rats. Behav. Brain Res..

[34] Humphrey NK (1974). Species and individuals in the perceptual world of monkeys. Perception.

[35] Fagot J, Martin-Malivel J, Depy D (1999). What is the evidence for an equivalence between objects and pictures in birds and nonhuman primates?. Cah. Psychol. Cogn..

[36] Tafazoli S, Di Filippo A, Zoccolan D (2012). Transformation-tolerant object recognition in rats revealed by visual priming. J. Neurosci..

[37] Minini L, Jeffery KJ (2006). Do rats use shape to solve "shape discriminations"?. Learn. Mem..

[38] Talpos JC, de-Wit L, Olley J, Riordan J, Steckler T (2016). Do wholes become more than the sum of their parts in the rodent (*Rattus Norvegicus*) visual system? A test case with the configural superiority effect. Eur. J. Neurosci..

[39] Truppa V, Spinozzi G, Stegagno T, Fagot J (2009). Picture processing in tufted capuchin monkeys (*Cebus apella*). Behav. Processes.

[40] Alemi-Neissi A, Rosselli FB, Zoccolan D (2013). Multifeatural shape processing in rats engaged in invariant visual object recognition. J. Neurosci..

[41] Shinskey JL, Jachens LJ (2014). Picturing objects in infancy. Child Dev..

[42] Brown MW, Warburton EC, Aggleton JP (2010). Recognition memory: Material, processes, and substrates. Hippocampus.

[43] Guderian S, Brigham D, Mishkin M (2011). Two processes support visual recognition memory in rhesus monkeys. Proc. Natl. Acad. Sci. U. S. A..

[44] Broadbent NJ, Gaskin S, Squire LR, Clark RE (2010). Object recognition memory and the rodent hippocampus. Learn. Memory.

[45] Clark RE, Zola SM, Squire LR (2000). Impaired recognition memory in rats after damage to the hippocampus. J. Neurosci..

[46] Alvarado MC, Rudy JW (1995). Rats with damage to the hippocampal-formation are impaired on the transverse-patterning problem but not on elemental discriminations. Behav. Neurosci..

[47] Rudy JW, Sutherland RJ (1989). The hippocampal formation is necessary for rats to learn and remember configural discriminations. Behav. Brain Res..

[48] Clark RE, West AN, Zola SM, Squire LR (2001). Rats with lesions of the hippocampus are impaired on the delayed nonmatching-to-sample task. Hippocampus.

[49] Winters BD, Saksida LM, Bussey TJ (2008). Object recognition memory: Neurobiological mechanisms of encoding, consolidation and retrieval. Neurosci. Biobehav. Rev..

[50] Tuscher JJ, Taxier LR, Fortress AM, Frick KM (2018). Chemogenetic inactivation of the dorsal hippocampus and medial prefrontal cortex, individually and concurrently, impairs object recognition and spatial memory consolidation in female mice. Neurobiol. Learn. Mem..

[51] Stackman RW (2002). Small conductance Ca^2+^-activated K^+^ channels modulate synaptic plasticity and memory encoding. J. Neurosci..

