# Supplementary figures and images for: Mice recognize 3D objects from recalled 2D pictures, support for picture-object equivalence
Source: Sci Rep. 2022 Mar 9;12:4184. doi: 10.1038/s41598-022-07782-4 (PMC8907285; doi:10.1038/s41598-022-07782-4)

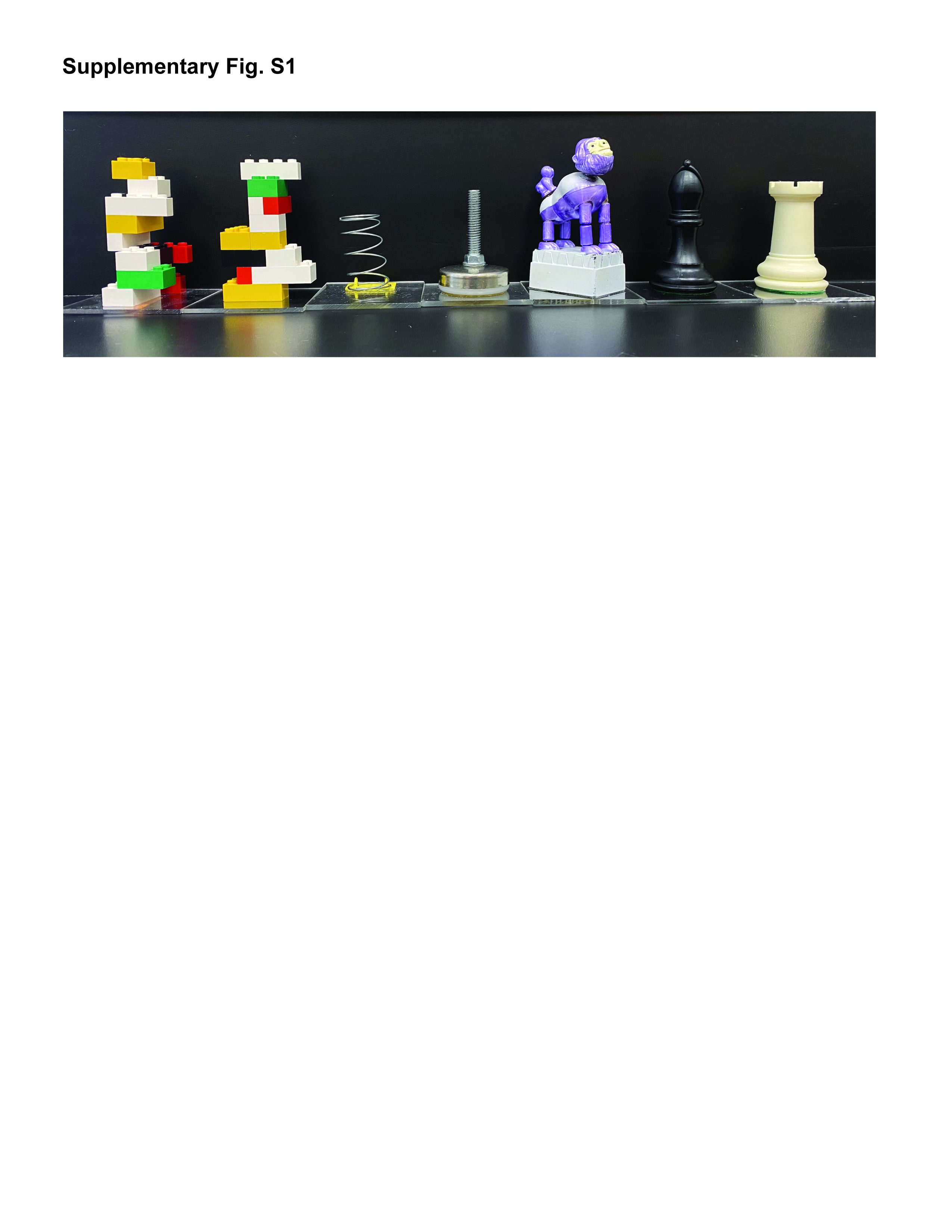

Supplement: Supplementary file 1 — Supplementary Information 1. [file 41598_2022_7782_MOESM1_ESM.tif]

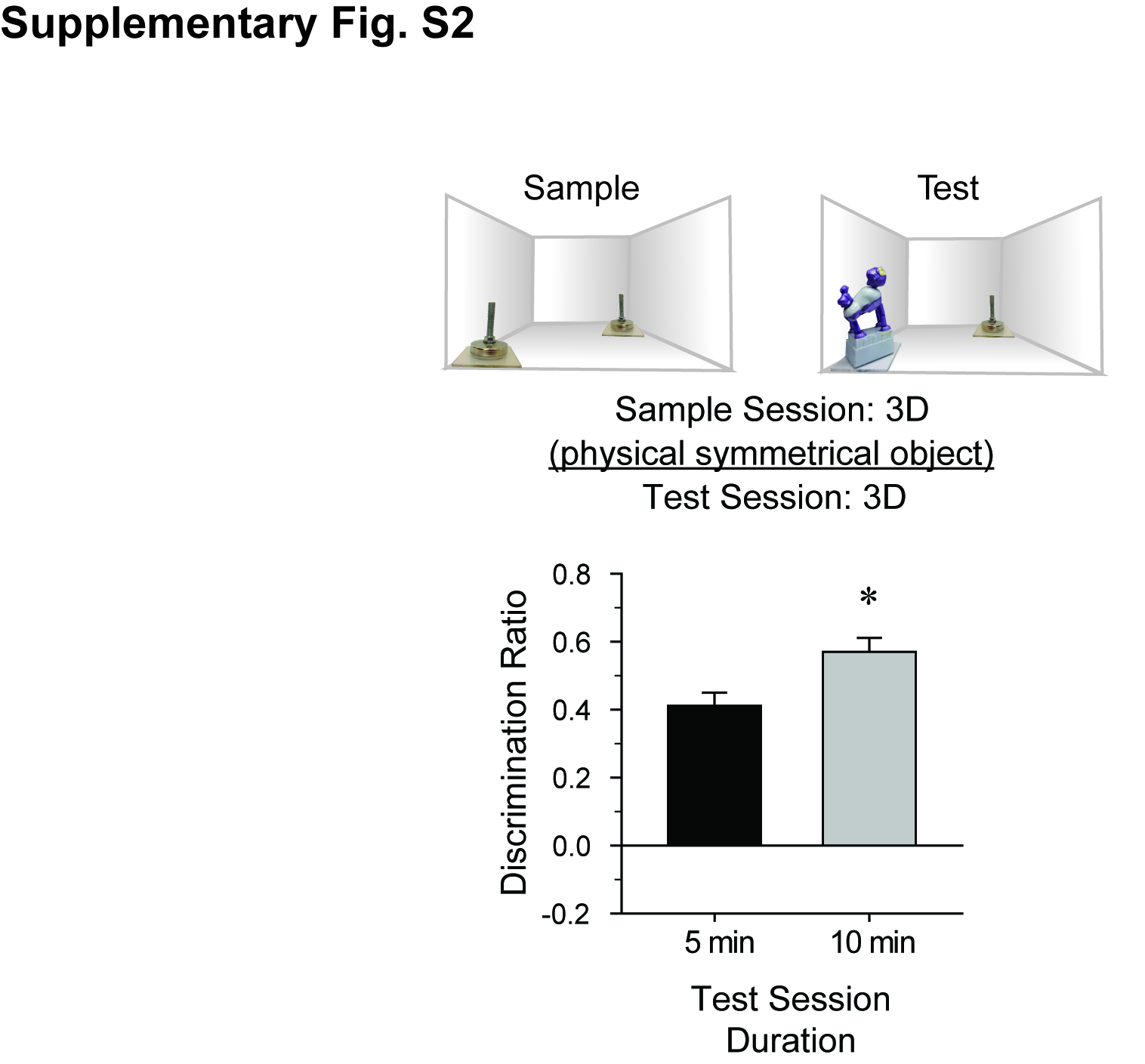

Supplement: Supplementary file 2 — Supplementary Information 2. [file 41598_2022_7782_MOESM2_ESM.tif]

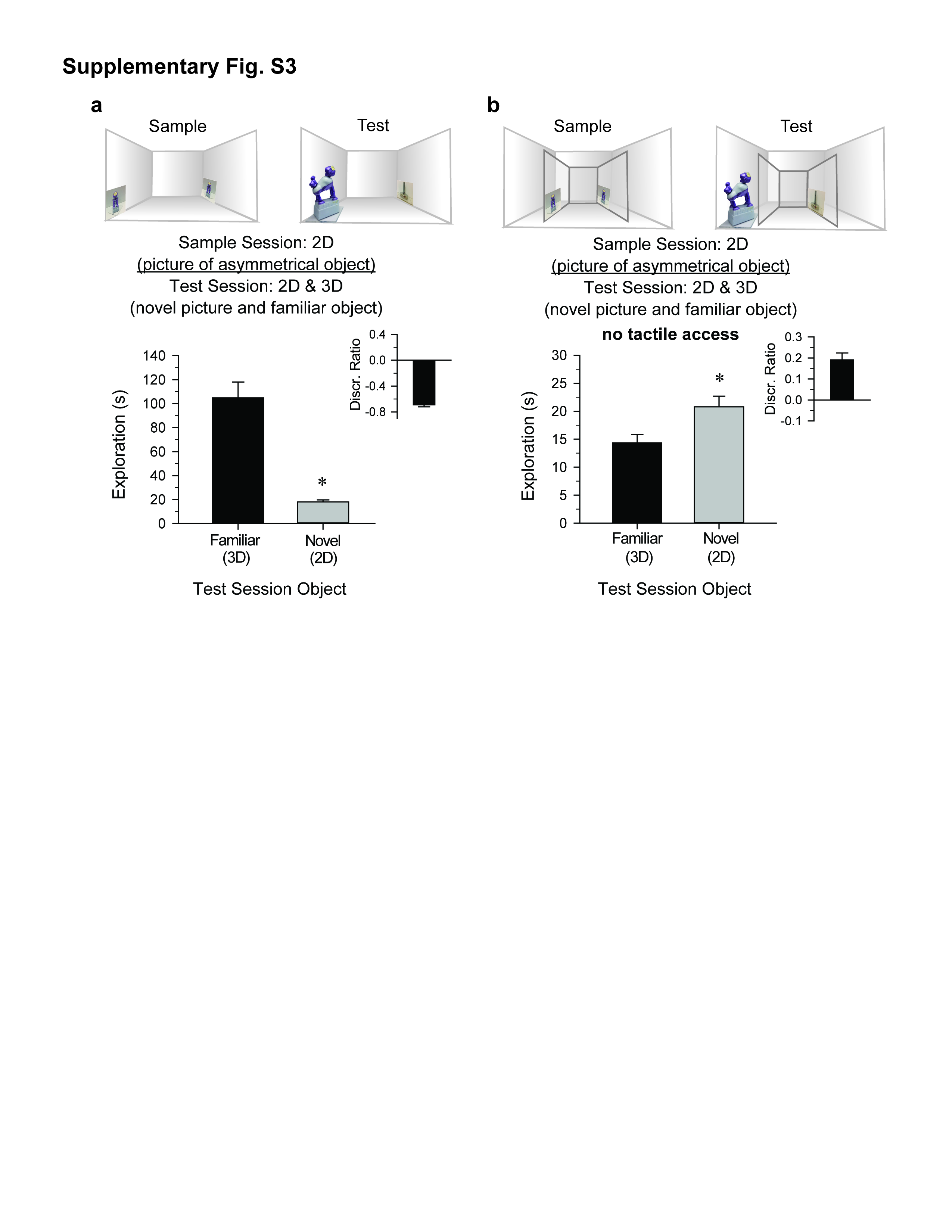

Supplement: Supplementary file 3 — Supplementary Information 3. [file 41598_2022_7782_MOESM3_ESM.tif]

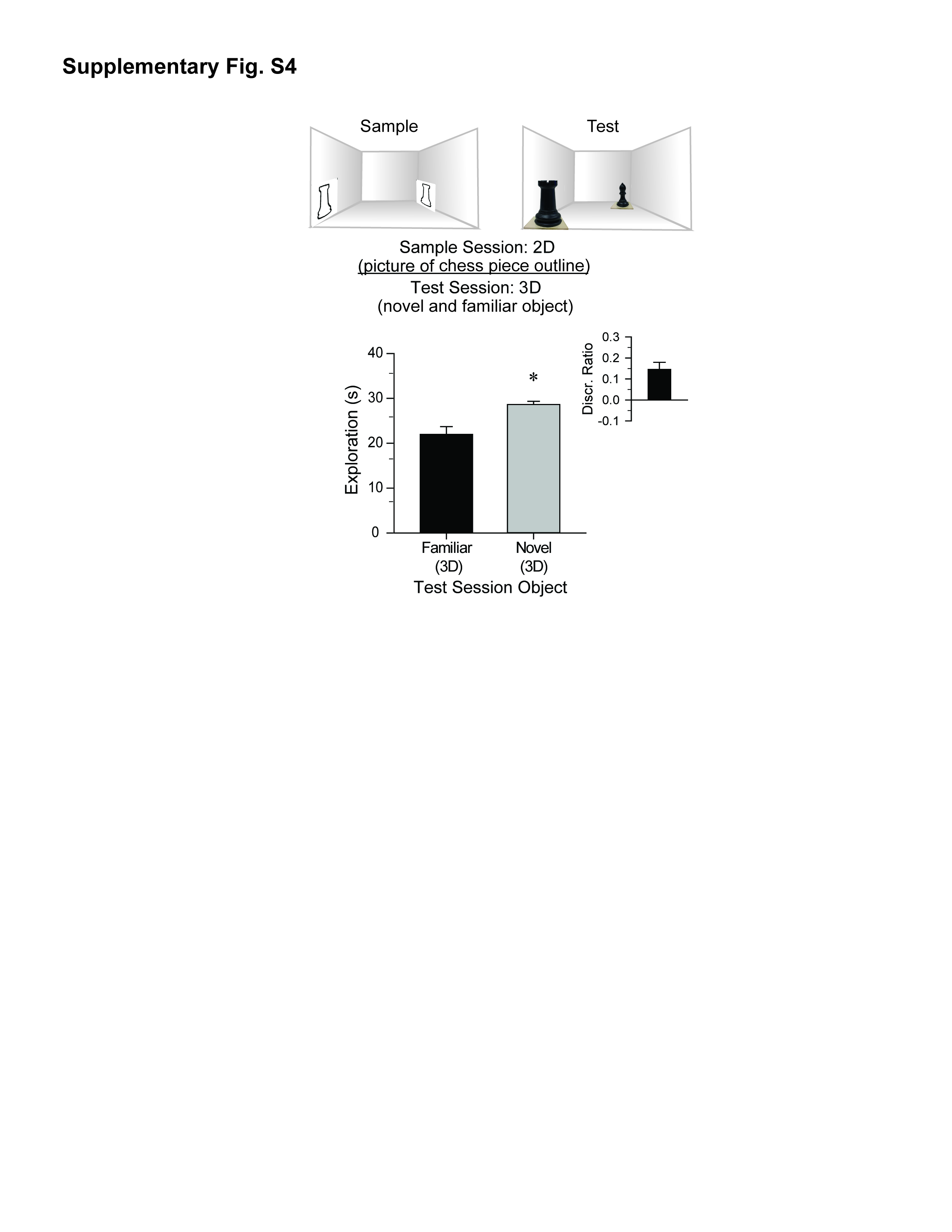

Supplement: Supplementary file 4 — Supplementary Information 4. [file 41598_2022_7782_MOESM4_ESM.tif]
