# Supplementary material for: Mice recognize 3D objects from recalled 2D pictures, support for picture-object equivalence
Source: Sci Rep. 2022 Mar 9;12:4184. doi: 10.1038/s41598-022-07782-4 (PMC8907285; doi:10.1038/s41598-022-07782-4)
Supplement: Supplementary file 5 — Supplementary Information 5. [file 41598_2022_7782_MOESM5_ESM.docx]

*Scientific Reports*

Article

Supplementary Figures

Mice recognize 3D objects from recalled 2D pictures, support for picture-object equivalence

Sarah J. Cohen^1, 2^, David A. Cinalli, Jr.^3^, Herborg N. Ásgeirsdóttir^2, 4^, Brandon Hindman^3^,

Elan Barenholtz^1, 3^, and Robert W. Stackman, Jr.^1, 2, 3, 4*^

Affiliations:

^1^ Center for Complex Systems & Brain Sciences, Florida Atlantic University, Boca Raton, FL 33431. USA.

^2^ Jupiter Life Science Initiative, Florida Atlantic University, John D. MacArthur Campus, Jupiter, FL 33458. USA.

^3^ Department of Psychology, Charles E. Schmidt College of Science, Florida Atlantic University, Boca Raton, FL 33431. USA.

^4^ FAU and Max Planck Florida Institute Joint Integrative Biology - Neuroscience Graduate Program, Florida Atlantic University, Jupiter, FL 33458. USA.

* To whom correspondence should be addressed:

Robert W. Stackman Jr., Ph.D., Florida Atlantic University, Jupiter Life Science Initiative, John D. MacArthur Campus, Jupiter, FL 33458. USA. email: [rstackma@fau.edu](mailto:rstackma@fau.edu)

Running title: Picture-object equivalence in mice

Keywords: equivalence; memory; hippocampus; picture recognition; object recognition; mice

**Supplementary Figures**

**
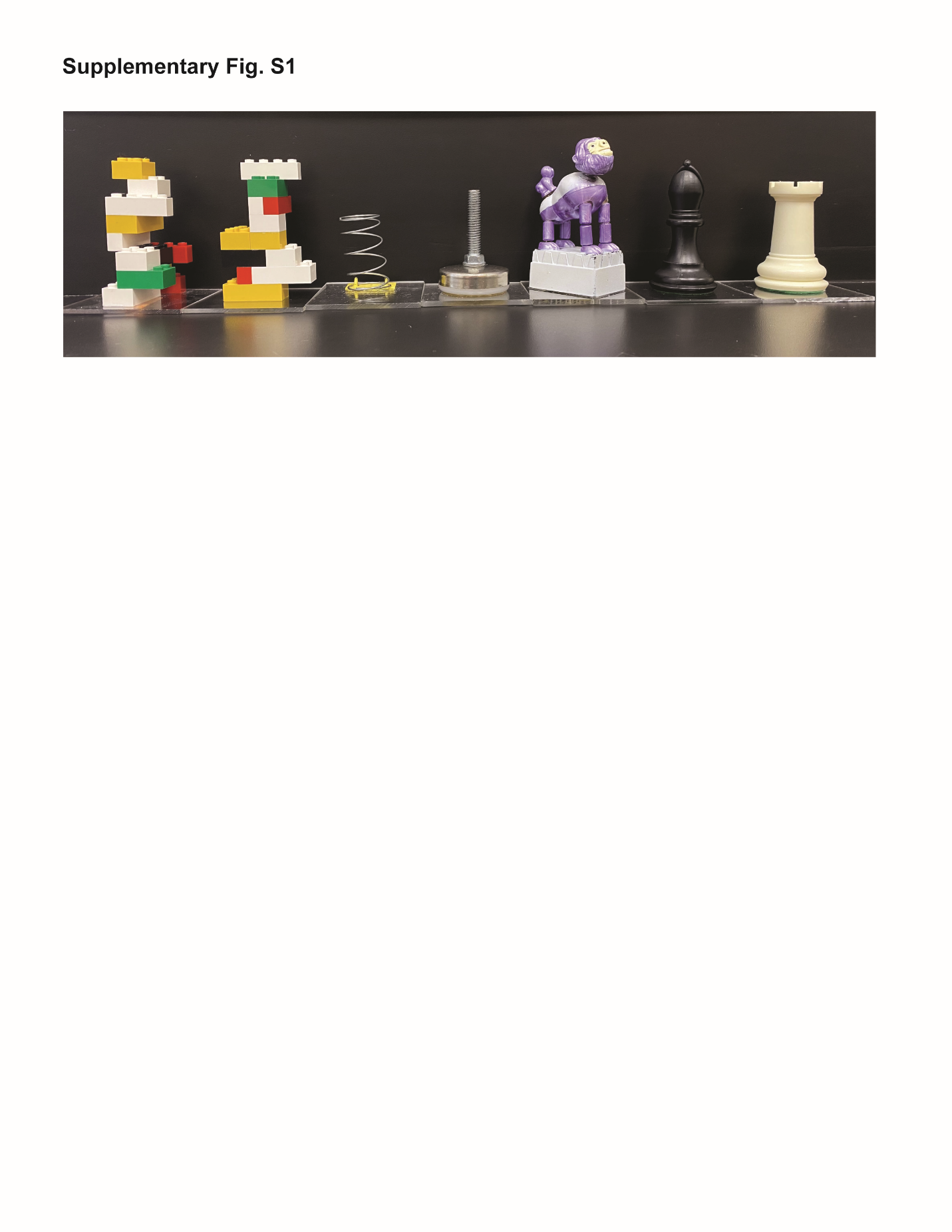
**

**Supplementary Fig. S1. 3D objects used in all experiments.** Close-up image of the 3D objects used in the experiments. From left to right, the objects are referred to as: LEGO (configuration 1), LEGO (configuration 2), spring, foot, monkey, bishop, rook. For each experiment described, the following stimuli were used during the sample and test session, respectively (as noted in the methods, where possible, stimuli were counterbalanced within each experiment). This photograph of the 3D objects was taken by S.J.C. using an iPhone 11 (Apple, Cupertino, CA). Respective 2D pictures of each object were laminated and affixed to the arena walls to serve as sample session stimuli, as shown in Fig 1. Specifically, in Fig. 1b: 2D foot, 2D foot & 2D monkey; Fig. 1c: 2D foot, 3D foot & 3D monkey; Fig. 1d: 2D monkey, 3D monkey & 3D foot; Fig. 2: 3D monkey, 3D monkey & 2D monkey; Fig. 3a: 2D spring, 3D spring & 3D foot; Fig. 3b: 2D LEGO (configuration 1), 3D LEGO (configuration 1) & 3D LEGO (configuration 2); Fig. 3c&d: 2D rook, 3D rook & 3D bishop; Fig. 4: 2D monkey, 3D monkey & 3D foot; Fig. 5: 2D monkey, 2D monkey & 2D monkey scrambled & 2D foot; Fig. 5b: 2D monkey scrambled, 3D monkey & 3D foot; Fig. 6a: 2D foot silhouette, 3D foot and 3D spring; Fig. 6b: 2D monkey silhouette, 3D monkey & 3D foot.

**
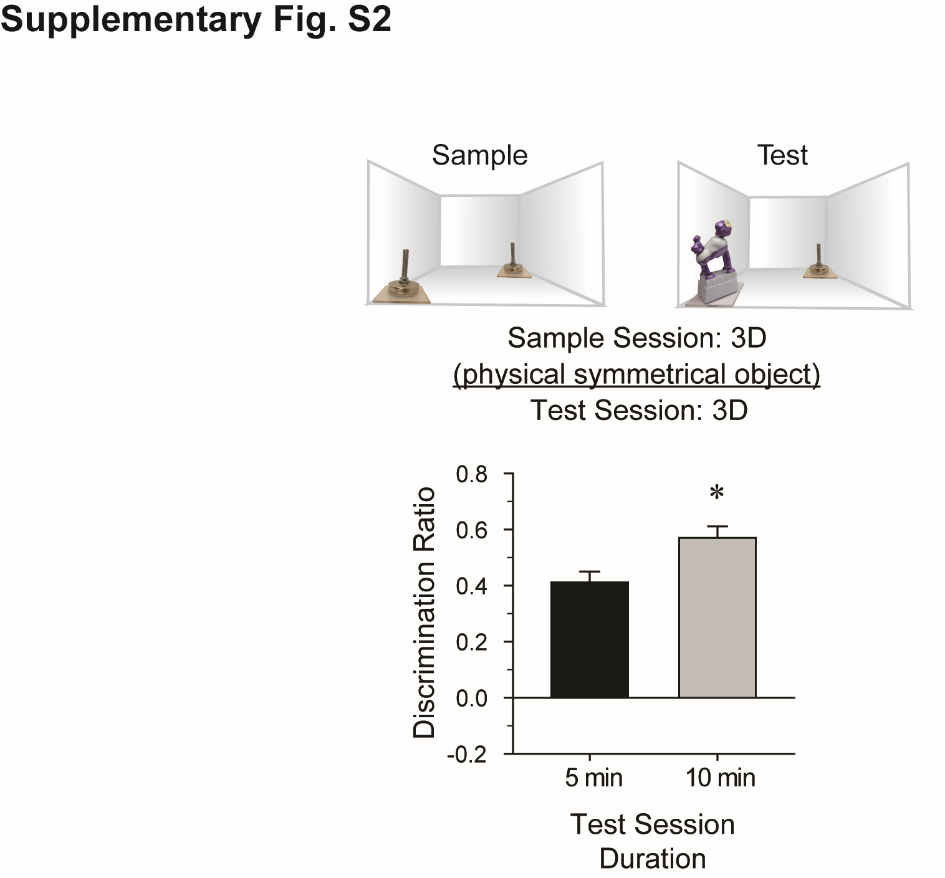
**

**Supplementary Fig. S2. Extending the duration of the traditional object recognition test session (3D stimuli for sample and test sessions) significantly increases discrimination between objects**

This experiment tested whether extending the duration of the test session would affect the expression of object discrimination in mice, as observed with picture-object equivalence. The sample and test sessions both consisted of 3D objects, with a 24 h delay between sessions. Mice that explored test session objects for a 5-min test session (n = 12) exhibited a significant discrimination between the familiar and novel objects [for data see 5]. Similarly, mice given a 10-min test session preferred the novel object [*t*(8) = -7.34, *P* < 0.01, *d* = 3.53], demonstrating clear discrimination between stimuli [*t*(8) = 13.94, *P* < 0.01, *d* = 4.64]. However, the mice given a 10-min test session displayed a significantly greater discrimination between objects than those given a 5-min test session [*t*(19) = -2.81, *P* = 0.01, *d* = 1.25]. These findings suggest that novelty-driven exploration follows a longer time course in mice than that described for other animals. *, *P* = 0.01 vs. 5 min test.

**
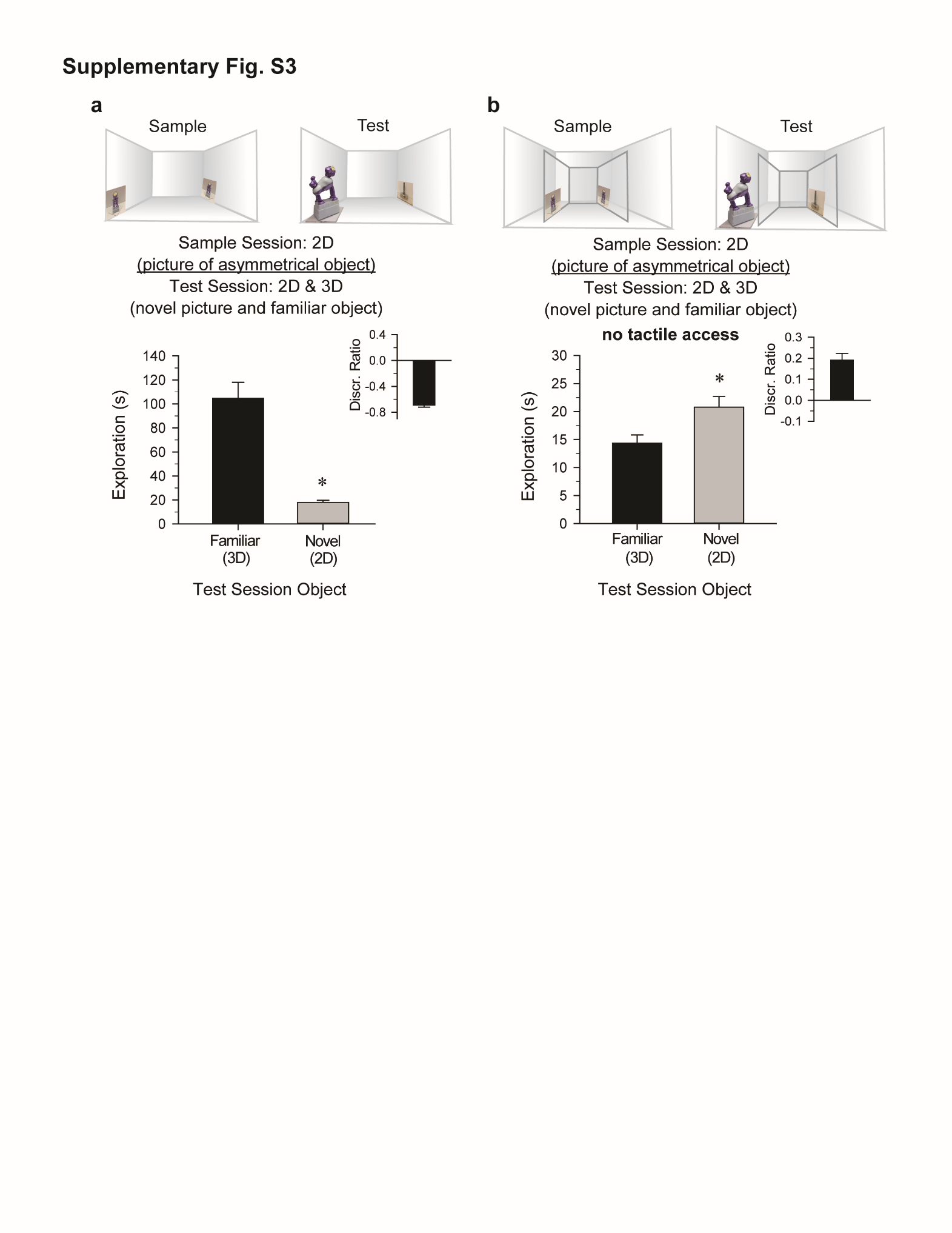
**

**Supplementary Fig. S3. Access to tactile information supersedes novelty preference when the novel stimulus is a 2D picture and the 3D object is “familiar”**

(**a**) We tested whether the preference for exploring the novel stimulus would extend to a more challenging condition in which the mice were presented with a novel 2D picture stimulus and a “familiar” 3D object. In this case, the novel 2D picture was more similar to the sample stimulus based on its physical nature alone, which may have led mice to explore it less than the “familiar” 3D object. However, if the mice truly formed 2D to 3D equivalence, then it was predicted that the novel 2D picture would be preferred over the “familiar” 3D stimulus. (**a)** Naïve mice (n = 10) explored 2D pictures during the sample session. Mice preferentially explored the "familiar" 3D object over the novel 2D picture during the test session 24 h after the sample session [*t*(9) = 7.02, *P* < 0.01, *d* = 2.92], presumably because 3D objects are more appealing to mice than 2D pictures [discrimination ratio (see *inset*), *t*(9) = -23.00, *P* < 0.01, *d* = 3.92]. (**b)** To test whether the substantial tactile information provided by the 3D object overrode the proclivity of mice to explore the novel 2D stimulus, we repeated the experiment, but the stimuli were placed outside of a clear Plexiglas insert within the arena to prevent access to tactile cues. This time, during the test session, the mice preferentially explored the *novel* 2D picture, over the “familiar” 3D object [*t*(9) = -5.50, *P* < 0.01, *d* = 1.20]. Inset, mice exhibited a significant discrimination of the novel 2D picture over the 3D “familiar” object [*t*(9) = 5.80, *P* < 0.01, *d* = 1.84]. This result indicates that when tactile information is unavailable, object exploration during the test session is guided by stimulus novelty rather than the physical characteristics of the objects. These results provide further support for the view that mice are capable of picture-to-object equivalence. *, *P* < 0.01 vs. the “familiar” 3D object.


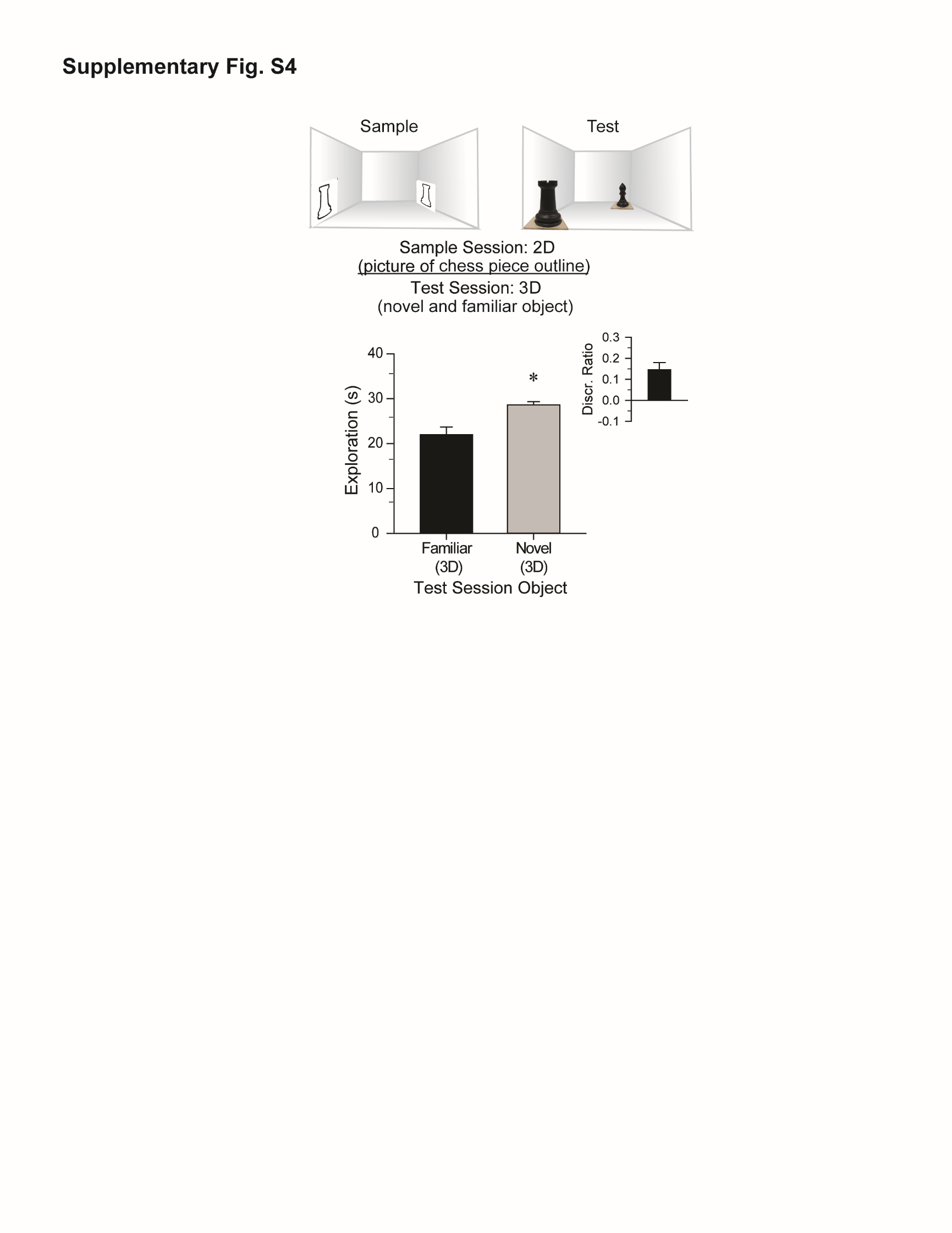


**Supplementary Fig. S4. Presentation of an Abstract Sample Item Does Not Affect the Recognition of a 3D Object from a 2D Picture**

To further test the notion that mice can correctly identify a novel object from a ‘familiar’ object seen only as an abstract depiction, we tested mice using an outline of the ‘familiar’ object during the sample session. Similar to the silhouette experiment (see Fig. 6b), mice that explored 2D pictures of object outlines of the rook chess piece during the sample session, preferentially explored the novel 3D object (bishop chess piece) during the test session, indicating recognition of the ‘familiar’ object [*t*(6) = -4.957, *P* < 0.01, *d* = 2.47]. The mice successfully discriminated between the novel and ‘familiar’ objects [*t*(6) = 4.432, *P* < 0.01, *d* = 1.98], which provides additional support that picture-object equivalence is not a perceptual ability beyond the capabilities of mice.
